# Transcription factors Rv0081 and Rv3334 connect the early and the enduring hypoxic response of *Mycobacterium tuberculosis*

**DOI:** 10.1080/21505594.2018.1514237

**Published:** 2018-09-26

**Authors:** Xian Sun, Lu Zhang, Jun Jiang, Mark Ng, Zhenling Cui, Juntao Mai, Sang Kyun Ahn, Jingqian Liu, Jinyu Zhang, Jun Liu, Yao Li

**Affiliations:** a State Key Laboratory of Genetic Engineering, Institute of Genetics, School of Life Science, Fudan University, Shanghai, China; b Department of Molecular Genetics, University of Toronto, Toronto, Canada; c Shanghai Key Laboratory of Tuberculosis, Shanghai Pulmonary Hospital, Medical School, Tongji University, Shanghai, China

**Keywords:** Rv0081, Rv3334, *Mycobacterium tuberculosis*, hypoxia, transcriptional factors

## Abstract

The ability of *Mycobacterium tuberculosis* (*M. tb*) to survive and persist in the host for decades in an asymptomatic state is an important aspect of tuberculosis pathogenesis. Although adaptation to hypoxia is thought to play a prominent role underlying *M. tb* persistence, how the bacteria achieve this goal is largely unknown. *Rv0081*, a member of the DosR regulon, is induced at the early stage of hypoxia while *Rv3334* is one of the enduring hypoxic response genes. In this study, we uncovered genetic interactions between these two transcription factors. RNA-seq analysis of Δ*Rv0081* and Δ*Rv3334* revealed that the gene expression profiles of these two mutants were highly similar. We also found that under hypoxia, Rv0081 positively regulated the expression of *Rv3334* while Rv3334 repressed transcription of *Rv0081*. In addition, we demonstrated that Rv0081 formed dimer and bound to the promoter region of *Rv3334*. Taken together, these data suggest that Rv0081 and Rv3334 work in the same regulatory pathway and that Rv3334 functions immediately downstream of Rv0081. We also found that Rv3334 is a bona fide regulator of the enduring hypoxic response genes. Our study has uncovered a regulatory pathway that connects the early and the enduring hypoxic response, revealing a transcriptional cascade that coordinates the temporal response of *M. tb* to hypoxia.

## Introduction

*Mycobacterium tuberculosis* (*M. tb*), the causative agent of tuberculosis (TB), is one of the most successful human pathogens. The World Health Organization (WHO) estimated that as much as one-third of the world’s population is infected with *M. tb*. In the majority of these individuals, *M. tb* establishes a latent, asymptomatic infection that can persist for decades, which is termed as the latent TB infection (LTBI) [1,2]. About 5–10% of LTBI will eventually develop active disease and host immunosuppression (e.g., HIV coinfection) markedly increases the risk of reactivation [3]. LTBI poses a major challenge to effective control of TB because of the difficulty in diagnosis and the fact that LTBI represents a huge disease reservoir. A mathematical model of TB suggests that interventions including new drugs aimed at people with LTBI would be the most effective means reducing the incidence and mortality associated with TB. [4]

*M. tb* persisters, which are characterized by reduced or altered metabolic activity and enhanced drug tolerance, are thought to be a major contributor of LTBI [5–8]. They are slowly replicating sub-populations that arise stochastically, presumably in response to the host microenvironment conditions. As such they are unresponsive to currently available antibiotics that typically target metabolically active cells [9–12], and these persisters are also thought to be responsible for disease relapse following chemotherapy [5,10,13,14].

Although the exact stimuli that cause *M. tb* to enter latency are unknown, a variety of studies from human, animals and *in vitro* conditions point to an intimate link between oxygen tension and the outcome of *M. tb* infection [5–7,15–17]. For example, *M. tb* resides within human granulomas during latent infection, which is considered to be a hypoxic environment due to the lack of endothelial and blood vessel markers [18,19]. Conversely, reactivation of LTBI in humans occurs most frequently in the upper lobes of the lungs, which are the most oxygenated regions of the body [7].

Larry Wayne and co-workers were the first to develop an *in vitro* model to mimic the hypoxic environment of the human granuloma [12,20,21]. In the Wayne model, a sealed, standing culture is incubated over an extended period while the bacteria deplete the available oxygen. The gradual depletion of oxygen leads to nonreplicating persistence states (NRP) with a concomitant shift in gene expression and metabolism. Bacteria in the NRP states also exhibit an increased tolerance to drugs. Using this system, it was shown that an early bacterial response (2 hr) was the coordinated upregulation of 48 *M. tb* genes under the control of two sensor kinases (DosS and DosT) and the response regulator (DosR), known as the DosR regulon. [22–24] It was subsequently shown that the DosR regulon is induced by other stress conditions including exposure to nitric oxide, [25] carbon monoxide, [26] SDS, [27] and low pH [28]. The DosR regulon is also induced upon *M. tb* infection of macrophages [29] and mice. [30,31]

The DosR system has been extensively studied with the presumption that it is essential for *M. tb* persistence. However, induction of the DosR regulon during hypoxia is transient, with most genes returning to baseline expression levels by 24 hr. [32] Another set of 230 genes induced by longer hypoxia exposure (7 days) was later identified. These genes are collectively known as the enduring hypoxic response (EHR) genes, and their expression is DosR-independent [32].

Despite the progress, many important aspects of genetic programs of *M. tb* in response to hypoxia remain unknown. In this study, we focused on two transcription factors, Rv0081 and Rv3334. *Rv0081* is a member of the DosR regulon and is induced at the early stage of hypoxia [23]. *Rv3334* is one of the EHR genes induced at the later stage of hypoxia [32]. We found that under hypoxic conditions, Rv0081 and Rv3334 work in the same regulatory pathway and that Rv0081 positively regulates the expression of *Rv3334*, which in return represses the *Rv0081* transcription. We also found that Rv3334 activates transcription of more than one-third of the EHR genes. Our studies have identified a genetic interaction that connects the early and the enduring hypoxic response, revealing a transcriptional cascade that coordinates the temporal response of *M. tb* to hypoxia.

## Results

### *Construction of ΔRv0081 and ΔRv3334 of* M. tb

Among the DosR regulon, *Rv0081* is the only gene predicted to encode a transcriptional regulatory protein besides *dosR* itself (DosR positively regulates its own transcription) [22–24]. Previous studies have confirmed that Rv0081 is a transcription factor that negatively regulates its own expression, [33] and ChIP-seq analysis has revealed >700 binding sites of Rv0081 in the *M. tb* genome [34]. However, the precise role of Rv0081 in the hypoxic response of *M. tb* is unclear. Rv3334 is a member of the MerR family of transcriptional regulators, and a recent study has found that Rv3334 is an auto repressor but positively regulates the expression of *kstR*, which encodes a transcription factor [35]. Cholesterol catabolism is essential for *M. tb* persistence [36,37] and KstR controls a large regulon mediating cholesterol degradation and lipid metabolism [36]. As such, Rv3334 may play an active role in the response of *M. tb* to hypoxia.

**Figure 1.**
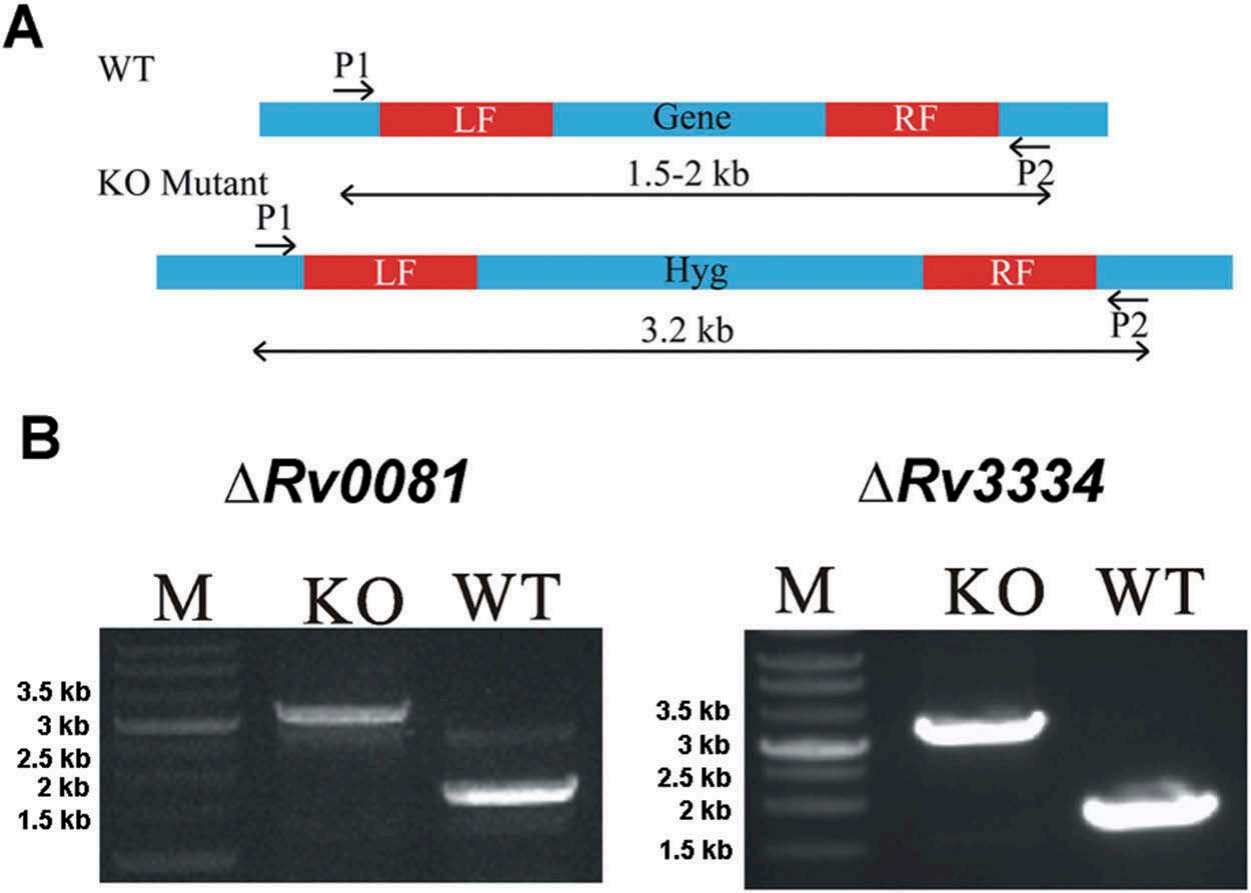
Construction of Δ*Rv0081* and Δ*Rv3334* of *M. tb*. (a) The coding sequence of *Rv0081* or *Rv3334* was replaced by a hygromycin resistance gene via homologous recombination using the left and right fragments (LF, RF). Primer pairs P1 and P2 were used to amplify the WT or the disrupted genes. The predicted size of PCR products containing *Rv0081* and *Rv3334* was 1.7- and 1.9- kb, respectively. The predicted size of PCR products for the knock out strains were 3.2 kb. (b) Agarose gel electrophoresis of the PCR products amplified from WT and knock out mutants. M: Marker, KO: knock out, WT: wild type.

To better understand the roles of Rv0081 and Rv3334 in hypoxic response of *M. tb*, we constructed two deletion mutants, Δ*Rv0081* and Δ*Rv3334*, of *M. tb* H37Rv using a transducing phage system [38]. The entire open reading frame (ORF) of each gene was replaced by a hygromycin resistance gene via homologous recombination using the fragments immediately up- and down-stream of the coding sequence (Figure 1(a)). The deletion mutants (Δ*Rv0081* and Δ*Rv3334*) were confirmed by PCR (Figure 1(b)) and RT-PCR analyzes. RT-PCR analysis showed that the expression level of *Rv0081* in Δ*Rv0081* and the expression level of *Rv3334* in Δ*Rv3334* were the same as the negative control (no cDNA template).

To complement the mutant strains, *Rv0081* and *Rv3334* were cloned into the pMV261 shuttle vector which contains an *hsp*60 promoter and the resulting constructs were introduced to the corresponding mutant strains, respectively. RT-PCR analysis showed that the expression levels of *Rv0081* and *Rv3334* in the complemented strains were increased 17.8- and 18.4-fold, respectively.

### ΔRv0081 and ΔRv3334 exhibited defective growth under hypoxia

**Figure 2.**
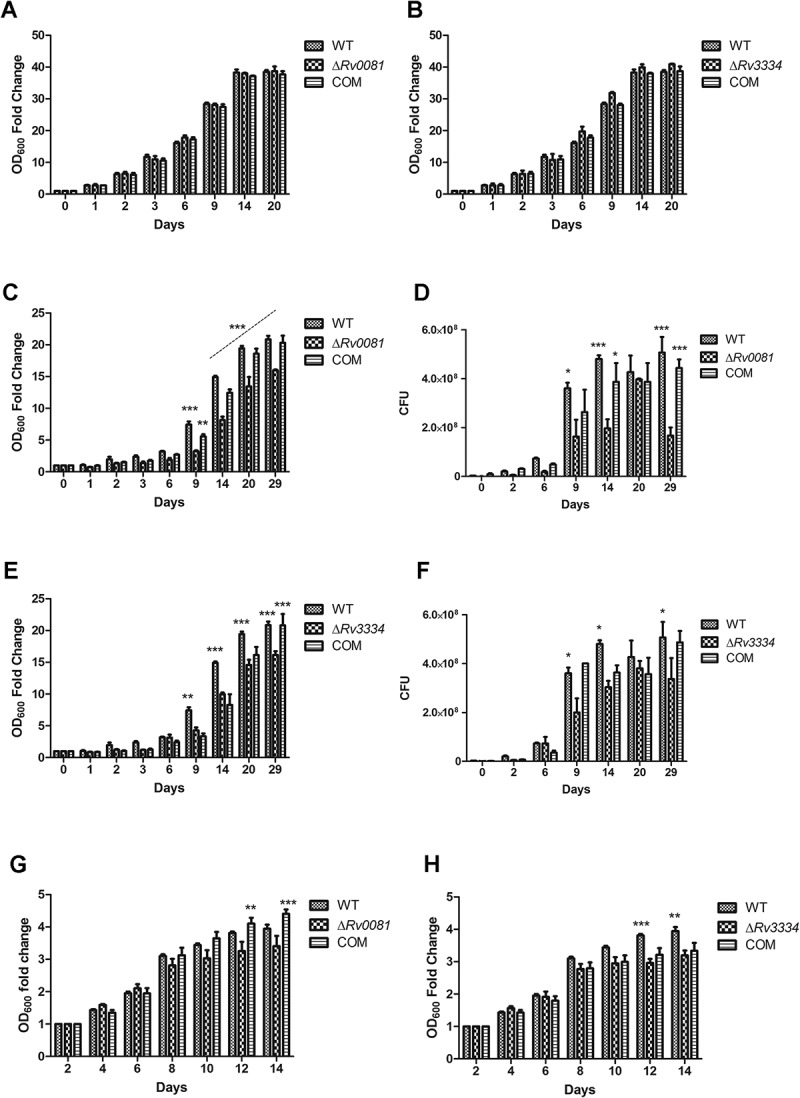
Δ*Rv0081* and Δ*Rv3334* exhibited defective growth under hypoxia. (a, c & d) Growth of Δ*Rv0081* under aerobic (a) and hypoxic conditions (c & d). (d) Cultures at the indicated time points from (c) were also plated to determine CFU. (b, e, & f) Growth of Δ*Rv3334* under aerobic (b) and hypoxic conditions (e & f). (f) Cultures at the indicated time points from (e) were also plated to determine CFU. (g) Growth of Δ*Rv0081* under aerobic conditions after recovery from 5-month hypoxia. (h) Growth of Δ*Rv3334* under aerobic conditions after recovery from 5-month hypoxia. Statistical analyzes (two-way ANOVA) were performed to compare all three groups. Significant differences between the mutant strain and WT or the complemented strain were indicated: *, *p *< 0.05; **, *p *< 0.01; ***, *p *< 0.001. In (c), the dashed line indicated that Δ*Rv0081* was significantly different (*p *< 0.001) to WT or the complemented strain at indicated time points (days 14, 20 and 29). COM: the complemented strains.

Under aerobic conditions, Δ*Rv0081* and Δ*Rv3334* grew equally well as the WT or the complemented strains (Figure 2(a,b)). However, they were defective in growth under hypoxia. Both mutants began to show reduced growth compared to the WT after 9 days under hypoxia, and this trend continued for the rest of time examined (29 days) (Figure 2(c–f)). For Δ*Rv0081*, complementation with *Rv0081* fully restored its growth under the hypoxic conditions (Figure 2(c,d)). Complementation of Δ*Rv3334* with *Rv3334* only partially restored its growth phenotype under hypoxia (Figure 2(e,f)).

To examine whether the mutant strains exhibit defective recovery from hypoxia, we set up another experiment in which cultures of the WT, Δ*Rv0081*, Δ*Rv3334*, and the complemented strains were incubated under hypoxia for 5 months. The hypoxic cultures were then collected and resuspended in fresh 7H9 media and their growths under aerobic conditions were compared. Under these conditions, Δ*Rv0081* did not show growth defect compared to the WT, although it did show reduced growth compared to the complemented strain at the later time points (Figure 2(g)). Δ*Rv3334* exhibited a slower recovery rate than the WT, but this phenotype was not restored in the complemented strain (Figure 2(h)).

### Rv0081 and Rv3334 are in the same regulatory pathway

To gain insight into the functions of Rv0081 and Rv3334, we performed RNA-seq and transcriptome analysis of Δ*Rv0081* and Δ*Rv3334*. Cultures of the WT, Δ*Rv0081*, and Δ*Rv3334* were grown under hypoxia for 7 days and then subjected to RNA-seq analysis. The same set of cultures grown under aerobic conditions for 7 days was also included. Since both mutant strains showed a similar growth pattern as the WT to this time point (7 days) under hypoxic or aerobic conditions (Figure 2), we chose these conditions to minimize the effect caused by differential growth among the strains. Expression of approximately 4,009 genes of *M. tb* was detected by RNA-seq.

Several important conclusions could be drawn from the comparative transcriptome analysis. First, Rv0081 and Rv3334 mainly function under hypoxic conditions as it was observed that the numbers of differentially expressed genes (DEGs, ≥2-fold, *Q *< 0.05) in Δ*Rv0081* and Δ*Rv3334* compared to the WT are much higher in the cultures grown under hypoxia than those grown aerobically. Comparing the hypoxic cultures of Δ*Rv0081* and the WT, a total of 605 DEGs were identified including 202 upregulated and 403 downregulated genes in Δ*Rv0081* (Dataset S1). In contrast, only 21 DEGs were detected between the cultures of Δ*Rv0081* and WT grown aerobically (Dataset S2). Similarly, a total of 762 DEGs including 365 upregulated and 397 downregulated genes were identified in the hypoxic culture of Δ*Rv3334* compared to that of the WT (Dataset S3). Only two genes (*Rv3269, Rv3378c*) were differentially expressed between the aerobic cultures of these strains.

**Figure 3.**
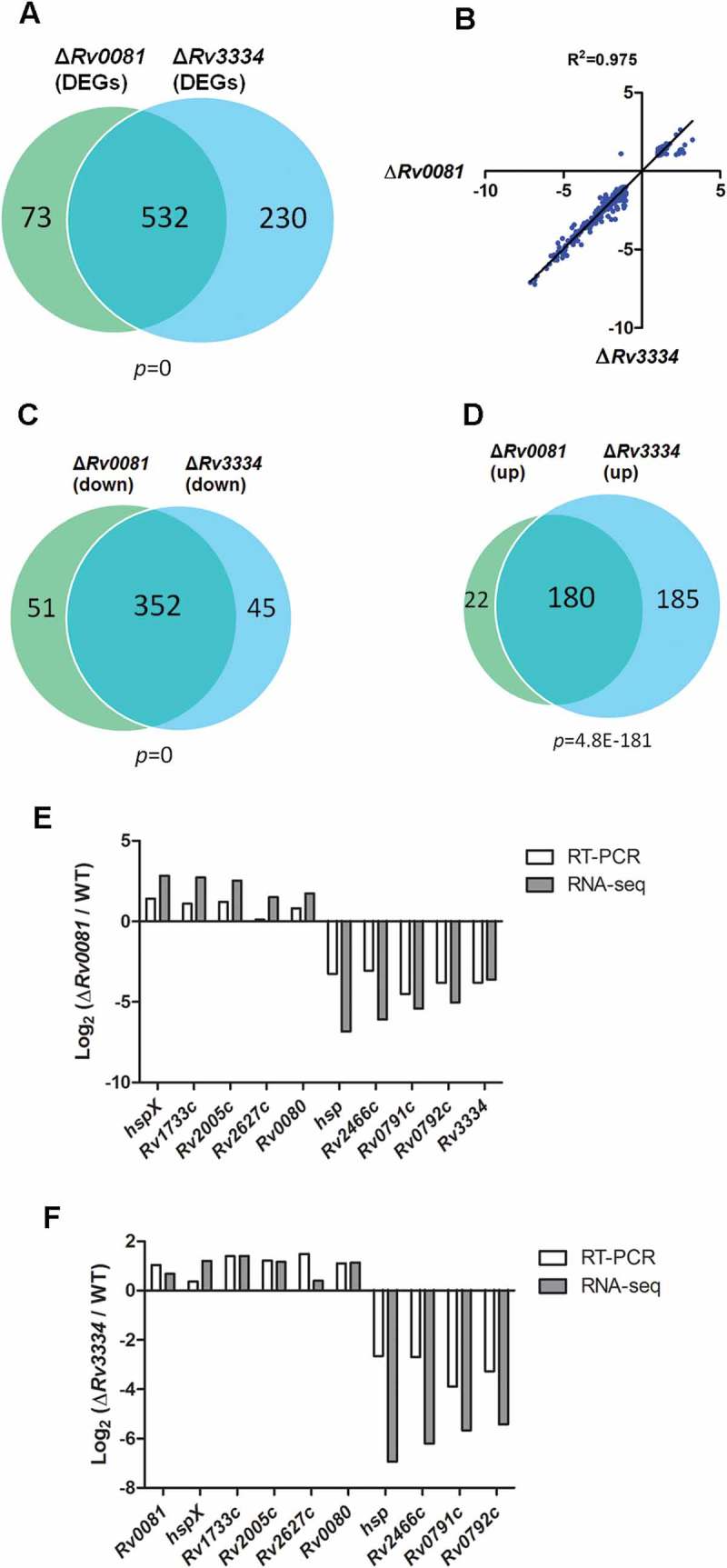
The gene expression profiles of Δ*Rv0081* and Δ*Rv3334* are highly similar. (a) Overlap of DEGs in hypoxic cultures of Δ*Rv0081* and Δ*Rv3334*, each compared to WT cultures under hypoxia. (b) The expression levels of 532 overlapped DEGs in Δ*Rv0081* and Δ*Rv3334* were compared and plotted. The correlation coefficient (*R* [2]) was calculated. (c) Overlap of the downregulated genes in hypoxic cultures of Δ*Rv0081* and Δ*Rv3334*, compared to WT hypoxic culture. (d) Overlap of the upregulated genes in hypoxic cultures of Δ*Rv0081* and Δ*Rv3334*, compared to WT hypoxic culture. The hypergeometric *p* values were calculated for each overlap analysis. (e & f) RT-PCR validation of RNA-seq data. Twelve genes including *Rv0081, Rv3334* and *sigA* were selected for RT-PCR analysis. The expression levels of each gene relative to *sigA* in hypoxic cultures were determined by RT-PCR and compared between WT and the mutant strains.

Secondly, the gene expression profiles of Δ*Rv0081* and Δ*Rv3334* are highly similar. Comparing the 605 DEGs of Δ*Rv0081* and 762 DEGs of Δ*Rv3334*, 532 were overlapped and the probability of the overlap by chance is nil (*p *= 0) (Figure 3(a)). Moreover, the expression levels of these overlapped DEGs were nearly identical in Δ*Rv0081* and Δ*Rv3334*. This is evident from the scatter plot using the expression level of individual DEGs in Δ*Rv0081* and Δ*Rv3334*, in which a nearly perfect correlation (*R^2^*=0.975) was observed (Figure 3(b)). Majority of the overlapped genes were those downregulated in Δ*Rv0081* and Δ*Rv3334* (Figure 3(c)) and their expression levels were nearly identical (Figure 3(b)), suggesting that Rv0081 and Rv3334 positively regulates almost the same genes. A lower level of, yet highly significant, overlap was observed between the genes upregulated in Δ*Rv0081* and Δ*Rv3334* (Figure 3(d)). Majority of the genes (180 out of 202) repressed by Rv0081 were also negatively regulated by Rv3334, and their expression levels in Δ*Rv0081* and Δ*Rv3334* were highly comparable (Figure 3(b)). The striking overlap in the gene expression profiles of Δ*Rv0081* and Δ*Rv3334* can be explained by the observation that there are regulatory interactions between Rv0081 and Rv3334. The expression of *Rv3334* was downregulated by 14.3 fold in Δ*Rv0081* and the expression of *Rv0081* was upregulated by 2.1 fold in Δ*Rv3334*, suggesting that Rv0081 activates the *Rv3334* transcription while Rv3334 represses the *Rv0081* expression.

RT-PCR analysis confirmed the above data, which showed that under hypoxia the expression level of *Rv3334* was 12.2 fold lower in Δ*Rv0081* than in WT, and that the expression level of *Rv0081* was 1.6 fold higher in Δ*Rv3334* than in WT (Figure 3(e,f)). Eight other genes (*hspX, Rv1733c, Rv2005c, Rv2627c, Rv0080, hsp, Rv2466c, Rv0791c, Rv0792c*), representing up- or down-regulated genes identified by RNA-seq analysis, were also subjected to RT-PCR analysis, and the results were in agreement with the RNA-seq data (Figure 3(e,f)).

Taken together, these data suggest that Rv0081 and Rv3334 work sequentially in the same regulatory pathway and that Rv3334 acts immediately downstream of Rv0081. Majority of the genes regulated by Rv0081 (87.9%) were also regulated by Rv3334 in the same direction (positive or negative regulation), suggesting that the regulatory role of Rv0081 is largely mediated through Rv3334.

### *Rv3334 is a bona fide EHR activator and Rv0081 represses transcription of* dosR-dosS

In *M. tb*, there are 230 DosR-independent EHR genes and regulatory proteins responsible for the induction of these genes are unknown [32]. Importantly, our transcriptome analysis suggests that Rv3334 is one of the regulators responsible for inducing the EHR genes as more than one-third (83 genes) of these genes were positively regulated by Rv3334 (*p *= 4.3E-29). Nearly identical set of the EHR genes (81 genes) were also activated by Rv0081, consistent with our suggestion that the positive regulation of EHR genes by Rv0081 is likely mediated through Rv3334.

We also analyzed hypoxia-induced genes in the WT cultures. A total of 259 genes were induced in hypoxic culture of WT compared to its aerobic culture (Dataset S4). Of these, 31 genes were among the previously identified EHR genes including *Rv3334* and the overlap is statistically significant (*p = *0.00003) [32]. Differences in culture conditions and detection methods (e.g., RNA-seq vs microarray) between our and previous studies may explain the relatively small number of overlap between these two dataset. Despite this, there are still significant overlaps between hypoxia-induced genes in the WT identified in current study and the genes positively regulated by Rv0081 and Rv3334. Of the 259 genes induced in the WT under hypoxia, 122 and 117 overlapped with the genes positively regulated by Rv0081 (*p *= 1.3E-58) and Rv3334 (*p *= 2.6E-54), respectively, with 116 being upregulated by both Rv0081 and Rv3334. Taken together, these data suggest that Rv3334 is responsible for the induction of a subset of the EHR genes.

We also found that Rv0081 negatively regulates transcription of *dosR-dosS* as the expressions of *dosR* and *dosS* were upregulated by 4.1- and 3.3-fold, respectively, in Δ*Rv0081*. Since DosR positively regulates *Rv0081* [23], the repression of *dosR* by Rv0081 provides a negative feedback mechanism. In contrast, the expressions of *dosR* and *dosS* were not significantly changed in Δ*Rv3334*, which is consistent with the notion that Rv3334 mainly functions in the later stage of hypoxia.

### *Rv0081 binds the promoter of* Rv3334

**Figure 4.**
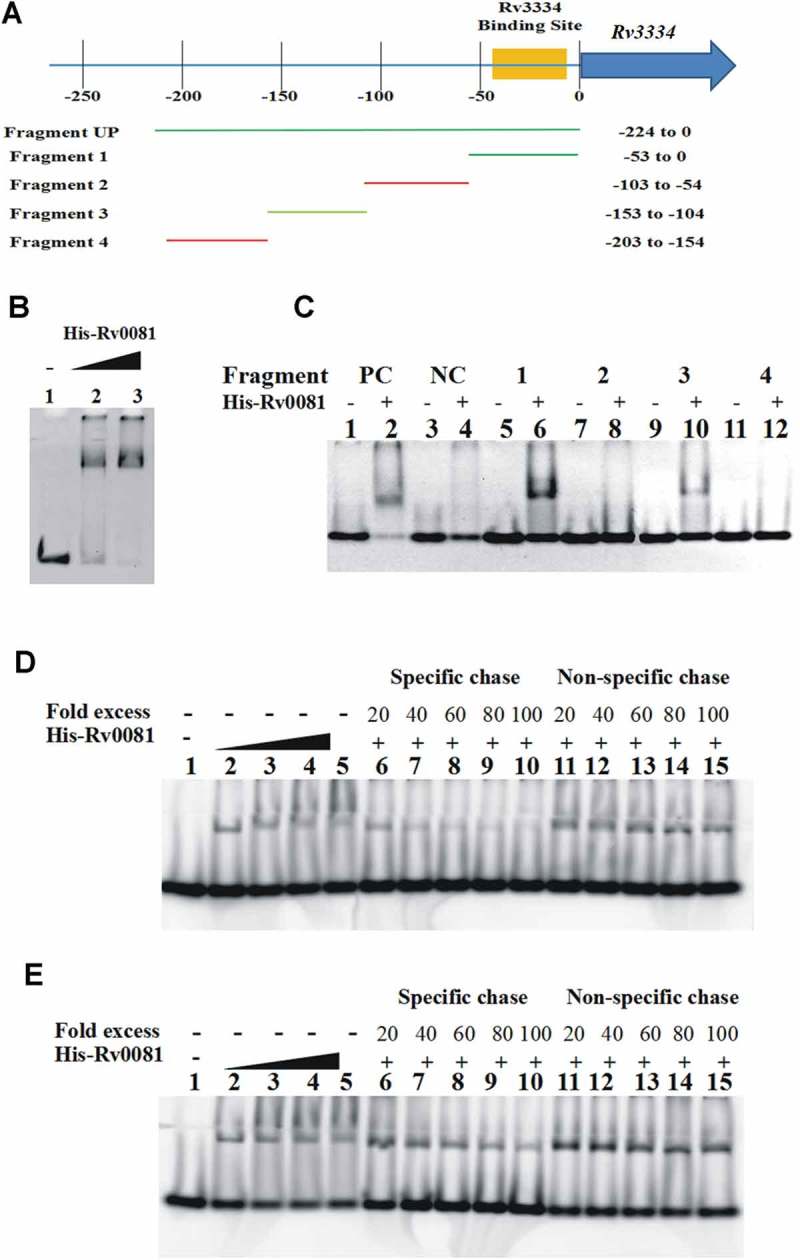
Rv0081 binds the promoter of *Rv3334*. (a) Schematic representation of different fragments upstream of *Rv3334* that were used in EMSAs to identify the binding site of Rv0081. DNA fragments are labeled as Fragment 1 to 4 and Fragment UP. Their corresponding positions with respect to start codon are indicated. The region marked by yellow shadow represents the 22-bp palindromic sequence, which is the binding site of Rv3334. (b) EMSA with Fragment UP. Lane 1: no protein; lane 2: 288 ng Rv0081 protein; lane 3: 576 ng Rv0081 protein. (c) EMSA with Fragment 1 to 4. For all reactions, 576 ng purified Rv0081 was used. Presence and absence of protein are indicated by (+) and (−), respectively. Lanes 1 & 2: positive control; lanes 3 & 4: negative control; lanes 5 & 6: Fragment 1; lanes 7 & 8: Fragment 2; lanes 9 & 10: Fragment 3; lanes 11 & 12: Fragment 4. (d & e) FAM-labeled Fragment 1 (d) and Fragment 3 (e) (10 pmol each) were applied in chasing system and visualized by fluorescence radiation. Lane 1: no Rv0081 protein; lanes 2 to 5: Rv0081 protein at 288-, 576-, 864- and 1152-ng were added; lanes 6 to 10: Rv0081 (1152 ng) and excessive unlabeled specific DNA were added; lanes 11 to 15, Rv0081 (1152 ng) and excessive unlabeled nonspecific DNA were added. PC: positive control, a 59-bp DNA fragment from the upstream region of *Rv0081*. NC: negative control, a 40-bp fragment from the upstream of *hycD.*

To determine whether regulation of the *Rv3334* transcription by Rv0081 is direct or indirect, His-tagged Rv0081 was overexpressed in *E.coli*, purified by column chromatography, and utilized in electrophoretic mobility shift assays (EMSAs). A double-stranded DNA fragment containing the 224 bp region upstream of the *Rv3334* start codon was amplified and used as the probe (Figure 4(a)). Results showed that Rv0081 bound this DNA fragment (Fragment Up) in a concentration dependent manner (Figure 4(b)).

To further map the binding site of Rv0081, the Fragment Up was divided into four DNA fragments each containing ~50 bp (Figure 4(a)), and their interactions with Rv0081 were examined by EMSAs. A 59-bp DNA fragment from the upstream region of *Rv0081* and a 40-bp fragment from the upstream of *hycD* were used as a positive and a negative control, respectively [33]. Results showed that Rv0081 bound DNA Fragment 1 (−53 to 0) and 3 (−153 to −104) from the *Rv3334* upstream region (Figure 4(c)). In contrast, Rv0081 did not bind Fragment 2 (−103 to −54) and 4 (−203 to −154). As expected, Rv0081 bound the upstream region of *Rv0081* (positive control) but did not interact with the upstream region of *hycD* (negative control) under the same experimental conditions (Figure 4(c)).

To determine whether the bindings of Rv0081 to Fragment 1 and 3 are sequence specific, we fluorescently labeled DNA probes (FAM-labeled Fragment 1 and 3) to use them in competition assays against unlabeled probes or a nonspecific DNA sequence. Binding of Rv0081 to FAM-labeled Fragment 1 was reduced in the presence of excessive unlabeled Fragment 1 but not with nonspecific DNA (Figure 4(d)). In contrast, the interaction between Rv0081 and Fragment 3 was not affected by either specific or nonspecific DNA, although a slight decrease of the binding was seen at very high concentration of specific DNA (80×and 100×) (Figure 4(e)). These results suggest that the main binding site of Rv0081 is present within 53 bp upstream of the *Rv3334* start codon.

**Figure 5.**
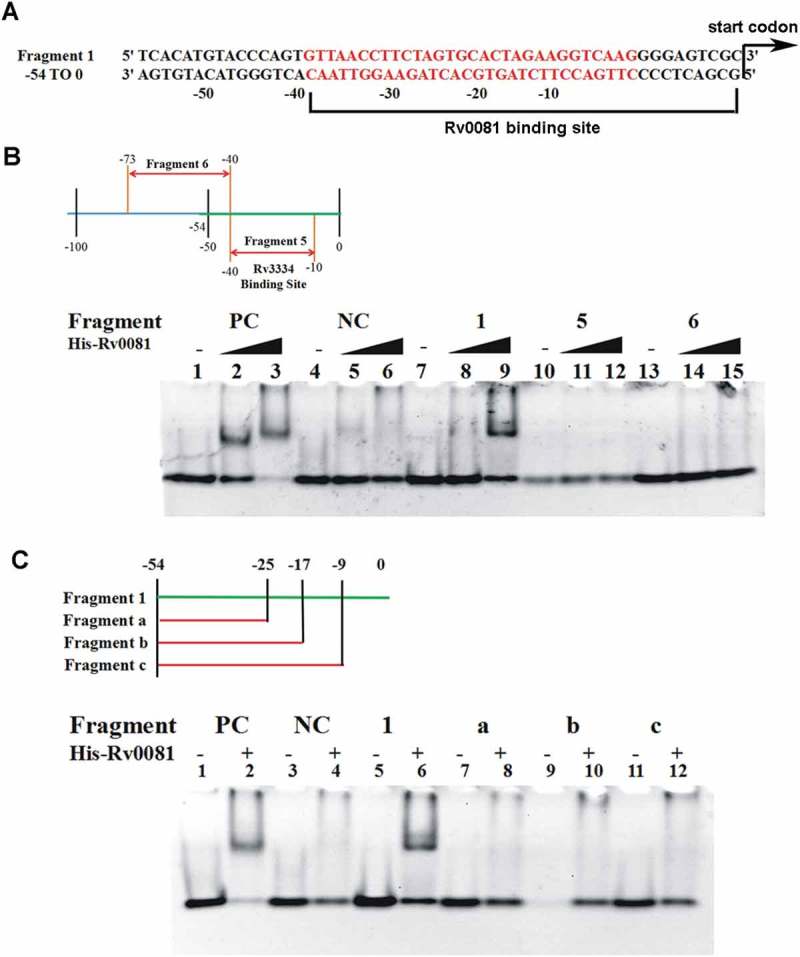
The first 10 bp and the 22 bp palindrome upstream of *Rv3334* are both required for the binding of Rv0081. (a) The 22-bp palindromic sequence in the upstream region of *Rv3334* was highlighted in red. The binding site of Rv0081 was indicated (−40 to −1). (b) EMSA of Fragment 5 (−40 to −10) and 6 (−73 to −40). Lanes 1 to 3: positive control DNA probe added with 0-, 288- and 576- ng Rv0081 protein, respectively; lanes 4 to 6: negative control DNA probe added with 0-, 288-, and 576- ng Rv0081, respectively; lanes 7 to 9: Fragment 1 added with 0-, 288-, and 576- ng Rv0081, respectively; lanes 10 to 12: Fragment 5 added with 0-, 288-, and 576- ng Rv0081, respectively; lanes 13 to 15: Fragment 6 added with 0-, 288-, and 576- ng Rv0081, respectively. (**c**) EMSA of smaller fragments: Fragment a (−54 to −25), b (−54 to −17), and c (−54 to −9). Lanes 1 & 2: positive control DNA added with 0- and 576- ng Rv0081 protein, respectively; lanes 3 & 4: negative DNA added with 0- and 576- ng Rv0081 protein, respectively; lanes 5 & 6: Fragment 1 added with 0- and 576- ng Rv0081 protein, respectively; lanes 7 & 8: Fragment an added with 0- and 576- ng Rv0081 protein, respectively; lanes 9 & 10: Fragment b added with 0- and 576- ng Rv0081 protein, respectively; lanes 11 & 12: Fragment c added with 0- and 576- ng Rv0081 protein, respectively. PC: positive control, a 59-bp DNA fragment from the upstream region of *Rv0081*. NC: negative control, a 40-bp fragment from the upstream of *hycD.*

Through sequence analysis, we noticed that the upstream region of *Rv3334* contained a 22 bp palindrome sequence (−35 to −14) (Figure 5(a)) which was previously shown to contain the binding site of Rv3334 (Figure 4(a)) [35]. To determine if this sequence is also sufficient for the Rv0081 binding, a DNA fragment (−10 to −40) containing the palindrome (Fragment 5) was synthesized and used in EMSAs, and the result showed that Rv0081 did not interact with this DNA fragment (Figure 5(b)). On the other hand, DNA fragments partially or entirely missing this palindrome (Fragment 6, −40 to −73; Fragment a, −25 to −54; Fragment b, −17 to −54) also failed to bind Rv0081 (Figure 5(b,c)). Similarly, no interaction was observed between Rv0081 and a DNA fragment containing the palindrome but missing the 10 bp immediately upstream of the *Rv3334* start codon (Fragment c, −9 to −54) (Figure 5(c)). Taken together, these data suggest that both the palindrome sequence and the first 10 bp upstream of the *Rv3334* start codon are required for the binding of Rv0081. In other words, the binding site of Rv0081 is at −40 to 0 upstream of *Rv3334* start codon (Figure 5(a)).

### *Rv0081 forms dimer* in vitro

**Figure 6.**
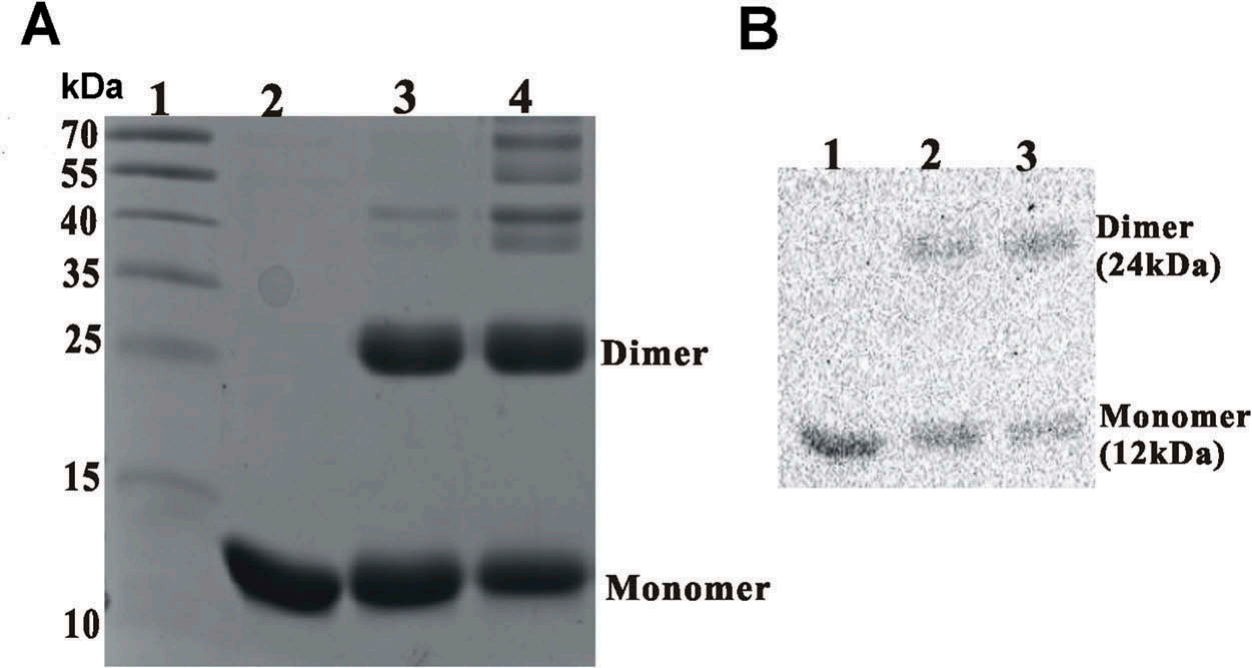
Rv0081 forms dimer. Purified Rv0081 protein was cross-linked by treatment with glutaraldehyde and then analyzed by SDS-PAGE and Coomassie blue staining (a) or Western blot (b). (a) lane 1: molecular weight marker; lane 2: Rv0081 without glutaraldehyde treatment; lanes 3 &4: Rv0081 treated with glutaraldehyde for 30 sec and 1 min, respectively. (b) Lanes 1: Rv0081 without glutaraldehyde treatment; lanes 2 & 3: Rv0081 treated with glutaraldehyde for 30 s and 1 min, respectively.

Based on the sequence analysis in NCBI-CDD, Rv0081 is a Smt/ArsR family transcription factor containing a helix-turn-helix DNA binding domain. Many of the known transcription factors with the domain form homodimers and bind to inverted repeats with two-fold symmetry. Given that the binding site of Rv0081 includes a 22-bp palindrome sequence, it is likely that Rv0081 also forms a dimer. To test this, purified Rv0081 protein was covalently cross-linked using glutaraldehyde and analyzed by SDS-PAGE. In the absence of cross-linking reagent, Rv0081 existed as a 12 kDa monomer. In the presence of 1% glutaraldehyde, the protein readily formed a homodimer of about 30 kDa (Figure 6(a)), which is confirmed by Western blot using the Rv0081 antibody (Figure 6(b)).

## Discussion

**Figure 7.**
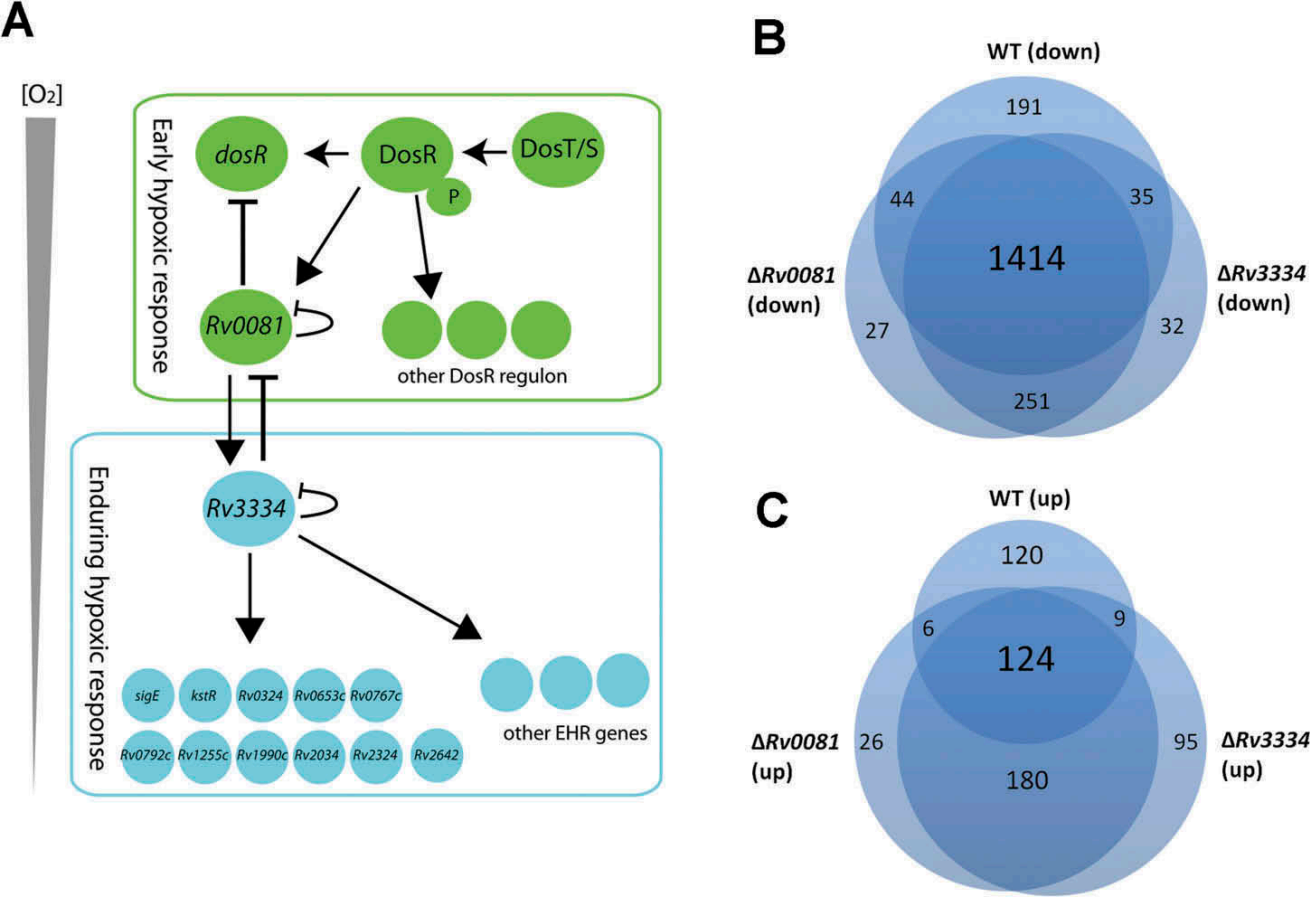
(a) Proposed model for the regulatory network of *M. tb* in response to hypoxia. See text for details. (b &c) Hypoxia induced changes of gene expression profiles in WT, Δ*Rv0081* and Δ*Rv3334*. Overlap of downregulated genes in hypoxic cultures of WT, Δ*Rv0081* and Δ*Rv3334* relative to their respective aerobic cultures (b). Overlap of upregulated genes in hypoxic cultures of WT, Δ*Rv0081* and Δ*Rv3334* relative to their respective aerobic cultures (c).

In this study, we discovered a regulatory pathway that connects the early and the enduring hypoxic response of *M. tb*. We found that Rv0081, a transcription factor that is induced at the early stage of hypoxia, positively regulated the expression of *Rv3334*, one of the EHR genes, and that gene regulation by Rv0081 is largely mediated through Rv3334. We also found that Rv3334 is an EHR regulator that activates a subset of the EHR genes. Based on our results and previous studies, we propose a model for the regulatory network of *M. tb* in response to hypoxia (Figure 7(a)). In this model, the early hypoxic response of *M. tb* is mediated by the DosR-DosS/T system, [23,39] which induces the expression of the DosR regulon including *Rv0081*. DosR positively regulates its own expression, which forms a positive loop that continuously increases the expression of *Rv0081* and other DosR-regulated genes. When intracellular Rv0081 accumulates and reaches a certain level, it negatively regulates the *dosR* expression thereby negating the early hypoxic response. At the same time, Rv0081 induces the expression of *Rv3334*. As the concentration of oxygen continues to decrease, Rv3334 reaches a threshold level and induces the expression of the EHR genes. Likewise, the induction of the EHR genes is controlled by a negative feedback mechanism in which Rv3334, once reaching a sufficient level, either directly or indirectly represses the expression of *Rv0081*.

Of the 230 EHR genes, 30 genes encode known or predicted regulatory proteins including *Rv3334* [32]. Interestingly, 11 of them were positively regulated by Rv3334, including *kstR, sigE, Rv0324, Rv0653c, Rv0767c, Rv0792c, Rv1255c, Rv1990c, Rv2034, Rv2324*, and *Rv2642*. (Figure 7(a)). Rv0081 also activates expression of these genes, and this regulatory activity is likely mediated through Rv3334. It is possible that these regulatory proteins work in sequential or parallel downstream of Rv3334, which together form the regulatory network responsible for the initial response and continuous adaptation to the hypoxic conditions. The transcriptional cascade allows *M. tb* to achieve a precise control of temporal and quantitative expression of specific genes in response to the gradual depletion of oxygen, and to reprogram the metabolism at different stages of hypoxia accordingly.

Consistent with the notion that hypoxia induces bacteriostasis, a large number of genes (1,684 genes, >40% of genome) were downregulated in the hypoxic culture of the WT compared to the aerobically grown WT culture (Dataset S4). Similar numbers of genes were also downregulated in the hypoxic cultures of Δ*Rv0081* (1,725 genes) and Δ*Rv3334* (1,722 genes) compared to their aerobically grown counterparts (Dataset S5 & S6). More than 80% of these downregulated genes (1,414 genes) overlap in all three strains (Figure 7(b)). In contrast, substantially more genes were induced by hypoxia in Δ*Rv3334* (408 genes) and Δ*Rv0081* (336 genes) than in WT (259 genes). In addition, <50% of these induced genes are overlapped among the three strains (Figure 7(c)). This suggests that a substantial number of different genes were induced by hypoxia in Δ*Rv3334* and Δ*Rv0081* compared to WT, which suggests a dysregulation of the normal response due to the disruption of the regulatory cascade in the mutant strains (Figure 7(a)). This dysregulation may provide an explanation for the defective growth of the mutant strains under hypoxia (Figure 2). Complementation of Δ*Rv0081* with *Rv0081* fully restored its growth phenotype under hypoxia (Figure 2(c,d)), whereas the phenotypes of Δ*Rv3334* were only partially restored by complementation of *Rv3334* (Figure 2(e,f,h)). This partial complementation is not due to a polar effect caused by the deletion of *Rv3334* since this gene is not part of an operon. One possible explanation is that the expression level of *Rv3334* needs to be precisely controlled. Since complementation with *Rv3334* in an overexpression plasmid (pMV261) resulted in excessive expression (18.4 fold), it failed to fully restore the mutant phenotype. Future studies using an integration vector and the native promoter to express *Rv3334* will help to test this hypothesis.

Sequence analysis suggests that Rv0081 is a member of the ArsR/SmtB family metal-dependent transcription factor. Members of this family typically function as transcriptional repressors, responding to divalent and multivalent heavy metal ions and controlling the expression of genes involved in metal uptake, efflux, sequestration, or detoxification [40]. In the presence of heavy metal ions, their DNA binding activity are inhibited, allowing derepression of target genes [40]. It is not clear whether Rv0081 is responsive to metal ions as the sequence of Rv0081 lacks a defined metal binding site. Previously, it was shown that Rv0081 binds an inverted repeat element in its own promoter and represses transcription [33]. Consistently, we found that Rv0081 forms dimer and binds the upstream region (−40 to −1) of *Rv3334* containing a 22 bp palindrome (−35 to −14). Interestingly, the 22 bp palindrome is the binding site of Rv3334 (Figure 4(a)), which negatively regulates its own expression [35]. Therefore, Rv0081 may activate the *Rv3334* expression by competing for the same binding site thereby preventing the auto repressor activity of Rv3334.

Intriguingly, sequence analysis suggests that Rv3334 is also a metal-dependent transcription factor, belonging to the MerR family [41]. MerR-like proteins generally function as repressors in the absence of metal ions and become activators allosterically upon metal binding [41]. Rv3334 binds its own promoter in the absence or presence of low concentrations of metal ions (Cu^++^ or Co^++^), but the DNA binding activity is abrogated in the presence of high concentrations of these metals [35]. This suggests that Rv3334 behaves more like a member of ArsR/SmtB family proteins at least when it regulates its own expression [35].

The fact that metal-sensing regulators such as Rv3334 and possibly Rv0081 are involved in hypoxia induced response suggests that exposure to divalent or heavy metal ions may be an important signal and/or stress condition that *M. tb* encounter in the host during persistent or latent infection. Consistent with this notion, a previous study found that macrophages increase copper uptake and deliver the available copper into phagosomes under hypoxic conditions [42]. The interplay of the complex signals from various stress conditions (hypoxia, nutrient deprivation, exposure to heavy metals) may ultimately define the *in vivo* host environment in which *M. tb* establishes the latent infection.

## Materials and methods

### Bacterial strains, media, and growth conditions

*M. tb* Δ*Rv0081* and Δ*Rv3334* were constructed from *M. tb* H37Rv using the TM4 phage-mediated transduction system [38] and described below. Mycobacteria were routinely grown in Middle brook 7H9 broth containing 0.2% glycerol, 10% OADC (oleic acid, bovine serum albumin, dextrose, and catalase; Difco) and 0.05% Tween 80 or 7H11 agar supplemented with 0.2% glycerol and 10% OADC at 37°C under aerobic conditions. Kanamycin and hygromycin, when appropriate, were added at 25 and 75 µg/ml, respectively. Cultures grown under these standard conditions were used as start cultures for all other experiments described below.

### Construction of ΔRv0081 and ΔRv3334

To generate Δ*Rv0081* and Δ*Rv3334* mutant strains, the ORF of *Rv0081* or *Rv3334* of *M. tb* H37Rv was replaced with a hygromycin-resistance cassette using TM4 phage-mediated specialized transduction [38]. The primer pairs used to PCR amplify the left and right fragments of *Rv0081* to generate the allelic exchange substrate were LF-0081 (5ʹ-GGCACTAGTCTGATT GTGAATTCGCGTCCG-3ʹ), LR-0081 (5ʹ-GTGAAGCTTCGCGTCTCCTGAGAGCTTC-3ʹ), RF-0081 (5ʹ-GGTTCTAGACGTAACGCCATGGGT-3ʹ) and RR-0081 (5ʹ-ATAGGTACC GACGTCGACGGGTA-3ʹ); and for *Rv3334* were: LF-3334 (5ʹ-CGCACTAGTGCCGCACTG ATAAATCGG-3ʹ), LR-3334 (5ʹ-TACAAGCTTGCGACTCCCCTTGACCTT C-3ʹ), RF-3334 (5ʹ-CGCTCTAGAAGGTCGCTGGGTACGC-3ʹ) and RR-3334 (5ʹ-TACGGTACCTGCGCA CCAAACTGTC-3ʹ). The resulting PCR products were digested with the restriction enzymes *Spe*I/*Hind*III and *Xba*I/*Kpn*I, respectively, and ligated with the 3650 bp fragment of pJSC284 pretreated with the same enzymes to generate pKO0081 and pKO3334 containing hygromycin resistance gene. After digestion with *Pac*I, pKO0081 and pKO3334 each were ligated with a *Pac*I digested phasmid phLR and packaged using the MaxPlax Lambda packaging extract (Epicentre), followed by transduction into *E. coli* NM759. The resulting phLR/pKO0081 and phLR/pKO3334 phasmid DNA from the transductants was electroporated into *M. smegmatis* mc^2^ -155 and plated for mycobacteriophage plaques at the permissive temperature of 30°C. A high-titre phage lysate, prepared from a temperature-sensitive phage plaque, was used to infect *M. tb* H37Rv at the non-permissive temperature of 37°C as described previously [38]. Hygromycin-resistant colonies were selected at 6-weeks post-transduction and confirmed by PCR analysis.

For complementation, the ORF of *M. tb Rv0081* or *Rv3334* was cloned into the pMV261 vector (Km^R^) to generate pRv0081 and pRv3334, which were then transformed into *M. tb* Δ*Rv0081* or Δ*Rv3334*, respectively, by electroporation. The complemented strains were confirmed by PCR and RT-PCR analysis.

### Growth under hypoxia

WT, Δ*Rv0081* and Δ*Rv3334*, and the complemented strains of *M. tb* were aerobically grown in 7H9 broth to OD_600_ ~ 0.5 at which point they were aliquoted (0.2 mL) to 2 mL screw-top tubes. For growth assay under hypoxia, the 2 mL tube was filled with 7H9 broth so that there was no headspace air as a source of oxygen. Sealed tubes were incubated without shaking at 37°C. Triplicate tubes of each strain were sacrificially opened to measure the OD_600_ at each time point. In some cases, the cultures were plated to determine the colony forming unit (CFU). Oxygen depletion was monitored using the oxygen indicator dye methylene blue as previously described. [21]

### Culture conditions and RNA extraction

*M. tb* WT, Δ*Rv0081* and Δ*Rv3334* were grown in 7H9 broth to log-phase and then transferred to 50 mL screw-top tubes filled with 7H9 media to a final OD_600_ of 0.05. The tubes were then sealed and incubated at 37°C with slow rotation (120 rpm). Cultures grown under these conditions for 7 days were collected as hypoxic cultures and subjected to RNA extraction. Aliquot cultures from each strain were grown in 50 mL 7H9 broth (OD_600_ = 0.05) under the standard (aerobic) condition with shaking for 7 days, which were then collected as the aerobic cultures and subjected to RNA extraction.

For RNA extraction, liquid cultures were pelleted and immediately resuspended in 1 mL of Trizol. Cells were disrupted by bead beating with glass beads by three 30s pulses. Chloroform (200 µl) was added after bead beating. The mixture was vortexed thoroughly for 30 s, and was centrifuged at 15,000 rpm for 15 min. The supernatant was transferred to new tubes and added with isopropyl alcohol with equal volume. The mixture was vortexed thoroughly for 30 s, and centrifuged at 15,000 rpm for 20 min. The pellet was washed with pre-cooled 75% ethyl alcohol twice. Each time the mixture was vortexed thoroughly for 30 s, and centrifuged at 15,000 rpm for 1 min. The pellet was dried and resuspended with RNase-free water.

### RNA-seq analysis

Purified RNA was used to construct cDNA library according to the TruSeq Stranded RNA LT Guide from Illumina. The concentration and size distribution of cDNA library was prepared by TruSeq Stranded Total RNA Library Prep kit and analyzed by Qubit 2.0 Fluorometer. The average library size was approximately 350 bp. High-throughput sequencing was carried out on an Illumina HiSeq 2000 system according to the manufacturer’s instructions (Illumina HiSeq 2000 User Guide) and 150-bp paired-end reads were obtained. The raw reads were filtered by Seqtk and then mapped to the *M. tb* H37Rv strain reference sequence (GenBank NC_018143.1) using Bowtie2 (version: 2–2.0.5) [43]. Counting of reads per gene was performed using HTSeq followed by TMM (trimmed mean of M-values) normalization [44,45]. Differentially expressed genes were defined as those with a false discovery rate <0.05 and fold-change >2 using the edgeR software [46].

### RT-PCR analysis

For RT-PCR validation of RNA-seq data, 1 µg RNA was reversed-transcribed to cDNA, which was then used as the template for RT-PCR analysis. The reaction mixture (10 µl) contains 1 × SYBR Green Mix, 10 µM each of the forward primer and reverse primer, 1 µl of 20 × diluted cDNA template. The following amplification program was used: polymerase activation at 95 °C for 10 s, 40 cycles of 95 °C for 15 s and 60 °C for 60 s, and the final step 95°C 15s. The primers for analyzing the selected genes were: *Rv0081* forward (5ʹ-CCGTTCGGTCGGTGAGTT-3ʹ), *Rv0081* reverse (5ʹ-AGCTGCTGGGACAGGTTCG-3ʹ); *Rv3334* forward (5ʹ-ACCCTGCTTGACCACGCTTCA-3ʹ), *Rv3334* reverse (5ʹ-GCTCATCGA GGTCGCTTTCC-3ʹ); *sigA* forward (5ʹ-GCAGCTGATGACCGAGCTTA-3ʹ), *sigA* reverse (5ʹ-TGGCTTCCAGCAGATGGTTT-3ʹ); *hspX* forward (5ʹ-CGAGGACGACATTAAGGCCA-3ʹ), *hspX* reverse (5ʹ-TGGACCGGATCTGAATGTGC-3ʹ); *Rv1733c* forward (5ʹ-CCTTGGGACTCTGGTTGAGC-3ʹ), *Rv1733c* reverse (5ʹ-CGATGTCGTGTTGCCAACTG-3ʹ); *Rv2005c* forward (5ʹ-TTCCGGGTTTGGACTTCTCG-3ʹ), *Rv2005c* reverse (5ʹ-CGACTTTTGCACCAGCTTCC-3ʹ); *Rv2627c* forward (5ʹ-GTCCCTGGCTGGTTTGTGTA-3ʹ), *Rv2627c* reverse (5ʹ-AATGTTCAGGCCGAGTTCGT-3ʹ); *Rv0080* forward (5ʹ-GGCGATCCGTCCAGTCAAT-3ʹ), *Rv0080* reverse (5ʹ-TCAAGGTCGTCGGCTTCGTA-3ʹ); *hsp* forward (5ʹ-CCATCGCGGCTTCCTATGAC-3ʹ), *hsp* reverse (5ʹ-TACTTCGTGATGGCGATGCG-3ʹ); *Rv2466c* forward (5ʹ-GCCGACGATCCATGTCAATG-3ʹ), *Rv2466c* reverse (5ʹ-GTAACCGAGGCATCCCAGAG-3ʹ); *Rv0791c* forward (5ʹ-GTTGGCTGCGACGGTTAATG-3ʹ), *Rv0791c* reverse (5ʹ-CGTCAGGAAGTGGTCGCATA-3ʹ); *Rv0792c* forward (5ʹ-CTGGTCACGGCCTATCTTCC-3ʹ), *Rv0792c* reverse (5ʹ-TAGCCTGTGCAATGCGTACA-3ʹ).

### Ethics statement

All of the animal procedures were approved by the local animal care committees at Fudan University (No. 15,001). All methods were performed in accordance with the relevant guidelines and regulations proved by Shanghai science and Technology Commission [No. SYXK(Hu) 2015–0006].

### Rv0081 purification and antisera preparation

The coding sequence of *Rv0081* was amplified by PCR using primers Pet-0081-F (5ʹ- ATGGAGTCCGAACCGCTGT-3ʹ) and Pet-0081-R (5ʹ-GCGGGTGGTTCGGCTAT-3ʹ) and the genomic DNA of *Mtb* H37Rv as the template. The fragment was then cloned into pET28b by *Bam*HI and *Hind*III digestion and ligation. To express the recombinant protein, the pET28b-Rv0081 construct was transformed into *E. coli* BL21(DE3)/pLysS cells and plated on LB (Luria-Bertani) agar containing kanamycin (50 µg/ml). After overnight incubation at 37°C, single colonies were randomly picked and grown in LB broth and subcultured to 1 L. To induce the expression of protein, *E. coli* BL21 cultures were grown at 37°C to mid-log phase and added with 1 mM IPTG for 4 hours. The culture was then collected and resuspended in lysis buffer (20mM Tris, pH 7.9, 500 mM NaCl, 5 mM imidazole), passed three times through a French press and centrifuged at 10,000 rpm for 30 min at 4°C. The collected supernatant was subjected to Ni-NTA His•Bind® Resin (Novagen) and purified following the protocol recommended by the manufacturer. Ultrafiltration was performed to remove imidazole from purified protein with dialysis buffer (20 mM Tris pH 7.9, 150 mM NaCl, 20% glycerol). Purified protein were frozen by liquid nitrogen and stored at −80°C.

To prepare the antisera against Rv0081, four C57BL/6 mice were immunized subcutaneously with a mixture containing 100 µl purified Rv0081 protein (0.5 μg) and 100 µl of Freund’s incomplete adjuvant (Sigma). The immunization procedure was repeated two more times (2 weeks apart). Two weeks after the last immunization, mice were sacrificed and their sera were collected.

### Electrophoretic mobility shift assays (EMSAs)

For electrophoretic mobility shift assays (EMSAs), the 224 bp region upstream of the *Rv3334* start codon was amplified by PCR using primers Fragment UP-F (5ʹ-CCGTTCTGCTAAAAGCCGTTAC-3ʹ) and Fragment UP-R (5ʹ-CCGTTCTGCTAAAAGCCG TTAC-3ʹ). For small DNA fragments (<100 bp), pairs of single-stranded oligonucleotides were annealed. The sequence of these fragments are Fragment 1 (5ʹ-CACATGTACCCAGTGTTAACCTTCTAGTGCACTAGAAGGTCAAGGGGAGTCGC-3ʹ), Fragment 2 (5ʹ-GTATGGGCTCACCGAGATCAG GCTCGTCACGATCGCCCGCACTGCTGGCGGCT-3ʹ), Fragment 3 (5ʹ-TGTCGATGGTTGTTCTCATCTGGTAACTCCTTTCCGCAGGCCGCAATTCAGCG-3ʹ), Fragment 4 (5ʹ-GGATGGTCA TAGTGGCGTCGGGCGCCAGGCCTGCGCGGGCACACGCGGTGCGG-3ʹ), Fragment 5 (5ʹ-GTTAACCTTCTAGTGCACTAGAAGGTCAAG-3ʹ), Fragment 6 (5ʹ-CGCCCGCACTGCTGG CGGCTCACATGTACCCAGT-3ʹ), Fragment a (5ʹ-CACATGTACCCAGTGTTAACCTTCTAG TGC-3ʹ), Fragment b (5ʹ-CACATGTACCCAGTGTTAACCTTCTAGTGCACTAGAAG-3ʹ) and Fragment c (5ʹ-CACATGTACCCAGTGTTAACCTTCTAGTGCACTAGAAGGTCAAGGG-3ʹ). DNA probes were incubated with purified Rv0081 protein at indicated amounts in a reaction buffer (20 mM KCL, 5% glycerol, 25 mM Tris-HCl, pH 8.0, 6 mM MgCl_2_, 0.5 mM EDTA) for 20 min at room temperature. The DNA-protein mixture was then loaded on 6% nondenaturing polyacrylamide gels and electrophoresed at 80 V for 2 hours at 4°C. The gels were stained with Gelstain DNA dye (TransGen Biotech) and visualized and captured using a UV capture system (Biorad). FAM (carboxyfluorescein)-labeled DNA fragment was annealed with complementary oligo synthesized by Invitrogen Life and used in competition experiments. Gels were detected by Typhoon system (GE healthcare). For EMSAs 30 pmol DNA probes were used in each reaction. For competition EMSAs, the amount of DNA probes was indicated by fold differences.

### Cross-linking of Rv0081

Glutaraldehyde (1%) was added to 1 µg of purified Rv0081 protein for 1 and 2 mins at room temperature. The mixtures were then heated at 65°C for 10 min to terminate the reaction. The samples were separated on 12% SDS polyacrylamide gel. Coomassie blue straining and Western blot were used to visualize the protein.

## Abbreviations

*M. tb*: *Mycobacterium tuberculosis*
TB: tuberculosis
WHO: World Health Organization
LTBI: latent TB infection
HER: enduring hypoxic response
EMSAs: electrophoretic mobility shift assays
ORF: open reading frame
