# Supplementary material for: Transcription factors Rv0081 and Rv3334 connect the early and the enduring hypoxic response of Mycobacterium tuberculosis
Source: Virulence. 2018 Sep 26;9(1):1468–82. doi: 10.1080/21505594.2018.1514237 (PMC6177252; doi:10.1080/21505594.2018.1514237)
Supplement: Supplemental Material [file kvir-09-01-1514237-s001.zip › Supplementary Legend.docx]

**Supplementary Legend**

**Dataset S1. The list of differentially expressed genes (≥2 fold) in hypoxic cultures of Δ*Rv0081* and WT.** FPKM (Fragments Per Kilobase of exon model per Million mapped reads) counts of each strain are shown. The *Q*-value is the adjusted *P*-value after considering FDR (False Discovery Rate). Q<0.05 is considered statistically significant.

**Dataset S2. The list of differentially expressed genes (≥2 fold) in aerobic cultures of Δ*Rv0081* and WT**.

**Dataset S3. The list of differentially expressed genes (≥2 fold) in hypoxic cultures of Δ*Rv3334* and WT.**

**Dataset S4. The list of differentially expressed genes (≥2 fold) in WT cultures grown under hypoxic and aerobic conditions.**

**Dataset S5. The list of differentially expressed genes (≥2 fold) in cultures Δ*Rv0081* grown under hypoxic and aerobic conditions.**

**Dataset S6. The list of differentially expressed genes (≥2 fold) in Δ*Rv3334* cultures grown under hypoxic and aerobic conditions.**
